# Supplementary material for: Natural Variation in Adventitious Rooting in the Alpine Perennial Arabis alpina
Source: Plants (Basel). 2020 Feb 3;9(2):184. doi: 10.3390/plants9020184 (PMC7076489; doi:10.3390/plants9020184)
Supplement: Supplementary file 1 [file plants-09-00184-s001.zip › plants-662724_SupplFigures_2ndSub.pdf]

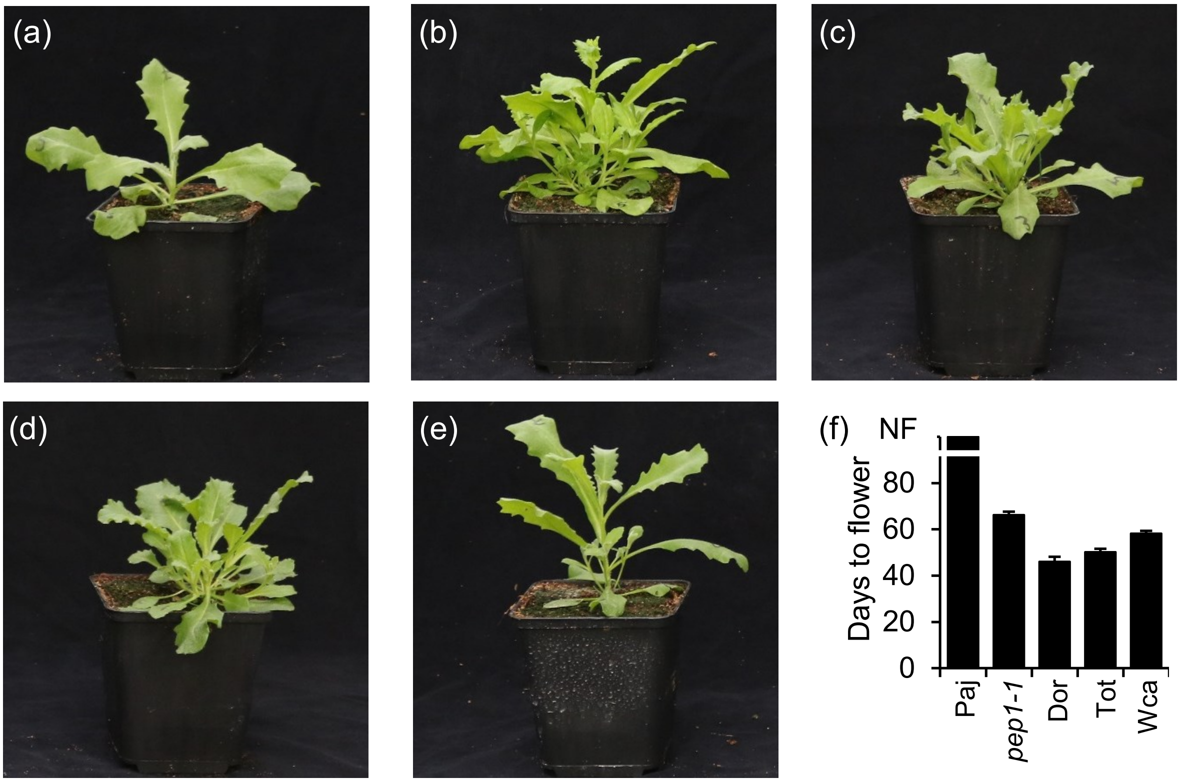

Figure S1. *A. alpina* accessions and mutants characterized for adventitious rooting. (a) Paj, (b) Dor, (c) Tot, (d) Wca and (e) *pep1-1* mutant grown in long days for 6 weeks. (f) Number of days to flower of *A. alpina* accessions and the *pep1-1* mutant in long days. Paj did not flower during the course of the experiment in the absence of vernalisation. NF: never flowered. n=9. Error bars indicate SD of three biological replicates.

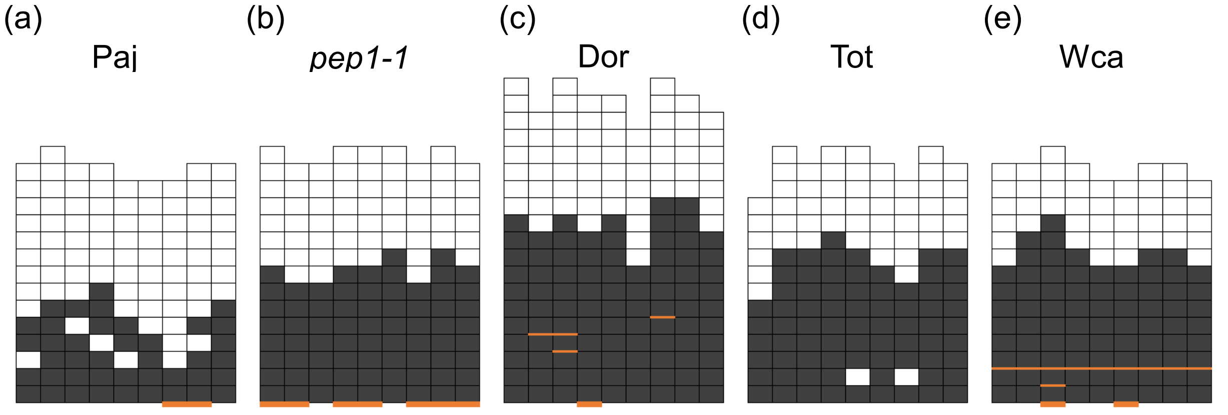

Figure S2. Adventitious rooting in *A. alpina* accessions. Each column represents a plant of (a) Paj, (b) *pep1-1* (c) Dor, (d) Tot and (e) Wca, with each box representing a leaf axil and the lines between boxes in a column representing an internode. The presence of branches (gray boxes) and adventitious roots (thick orange lines in a column) were scored in six-week old LD-grown plants. The thick orange lines at the bottom represent adventitious roots on the hypocotyl.

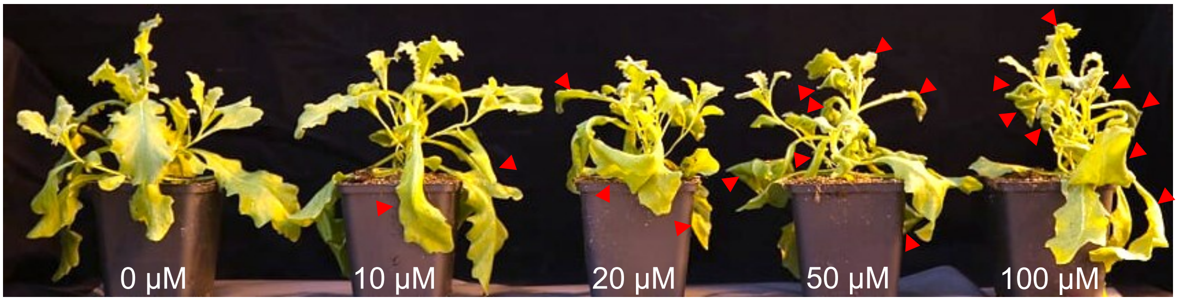

Figure S3: Leaf curling in response to different concentrations of 1-NAA. Six-week old plants were sprayed with 0, 10, 20, 50 and 100 µM 1-NAA. Plants from the accession Paj were scored one week after foliar spray. Curled leaves are shown with red triangles.

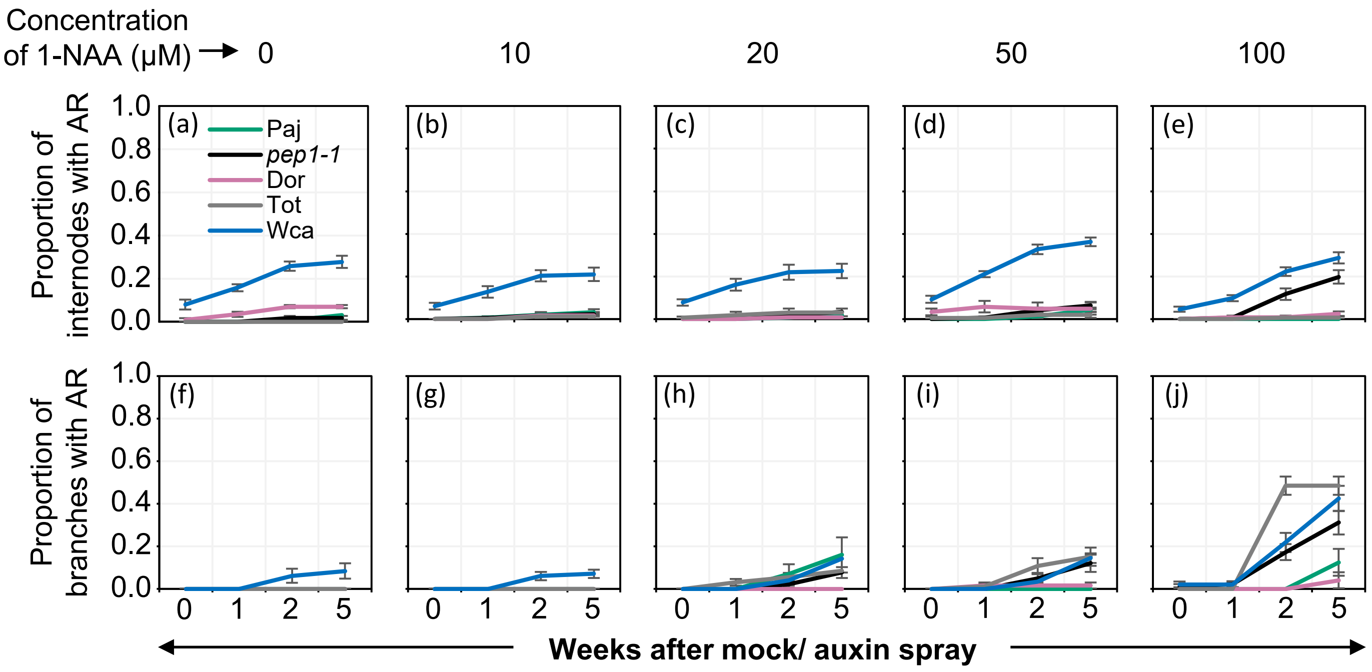

Figure S4. Auxin spray induces adventitious roots in a dosage- and genotype- dependent manner. Paj, *pep1-1*, Dor, Tot and Wca were sprayed after six weeks in LD with 0, 10, 20, 50 and 100 µM 1-NAA (n=9). Plants were scored before spray and 1, 2 and 5 weeks after auxin spray. (a) Proportion of internodes on the main stem and (b) axillary branches occupied with adventitious roots after 1-NAA spray relative to before spray. The characterization and the statistical analyses to understand the effect and interaction of genotype, age, concentration on adventitious root production are tabulated in Tables S7, S8 and S9.

Table S1. Significance test for the transcript levels of *AaGH3.3* between *A. alpina* genotypes. Summary data for an ANOVA and the following Tukey's multiple comparison post-hoc test for pairwise comparison of the expression of *AaGH3.3* in *A. alpina* accessions and the *pep1-1* mutant. The values with red font denote significant values and comparisons.

	Df	F value	p
**Name**	4	36.33	6.28e-06
**Residuals**	10		

Comparison	p
**Paj-Dor**	0.114
***pep1-1*-Dor**	0.075
**Tot-Dor**	0.970
**Wca-Dor**	0.000
***pep1-1*-Paj**	0.999
**Tot-Paj**	0.274
**Wca-Paj**	0.000
**Tot-*pep1-1***	0.187
**Wca-*pep1-1***	0.000
**Wca-Tot**	0.000

Table S2. Significance test for the transcript levels of *AaGH3.6* between *A. alpina* genotypes. Summary data for an ANOVA and the following Tukey's multiple comparison post-hoc test for pairwise comparison of the expression of *AaGH3.6* in *A. alpina* accessions and the *pep1-1* mutant. The values with red font denote significant values and comparisons.

	Df	F value	p
**Name**	4	137.4	1.08e-08
**Residuals**	10		

Comparison	p
**Paj-Dor**	0.000
***pep1-1*-Dor**	0.000
**Tot-Dor**	0.000
**Wca-Dor**	0.001
***pep1-1*-Paj**	0.007
**Tot-Paj**	0.000
**Wca-Paj**	0.000
**Tot-*pep1-1***	0.000
**Wca-*pep1-1***	0.000
**Wca-Tot**	0.781

Table S3. Significance test for the endogenous IAA levels between *A. alpina* stems. Summary data for an ANOVA and the following Tukey's multiple comparison post-hoc test for pairwise comparison of the levels of free endogenous IAA levels in the main stem of 6-week-old *A. alpina* accessions and the *pep1-1* mutant.

	Df	F value	p
**Ecotype**	4	1.51	0.27
**Residuals**	10		
